# Treatment of Carbuncle

**Published:** 1855-03

**Authors:** 


TREATMENT OF CARBUNCLE.

*General Treatment.*—Three or four different plans of treatment are in vogue in the Hospitals for the treatment of carbuncle.

1st. By stimulants, full diet, quinine, ammonia, etc.

2d. By opium, freely used, and generally combined with tonics.

3d. A more expectant plan, by salines, low diet, etc., in the first stage, and the moderate use of tonics afterwards. In all these, purgatives, of course, form a part.

One of the largest carbuncles which we have ever seen, occurred to a tailor, from Hampton Court, who was treated by Mr. De Morgan, in the Middlesex Hospital, about two years ago. The case was an extremely severe one, the depression of the vital powers being such that, for more than a fortnight the result seemed very doubtful. The very free use of opium, in combination with liquor cinchona, appeared to answer an excellent end, and the man ultimately recovered. We have not, however, seen the opiate plan tried on a sufficiently large scale to justify an opinion as to its general merits, the method by stimulants being by far the most frequently adopted. Mr. Lloyd, at St. Bartholomew's, is the surgeon who is, perhaps, the most sparing of any in the use of stimulating remedies; and we certainly have not observed that his cases do any worse than those of others. In the early stages, Mr. Lloyd generally prescribes salines and fever diet; unless, indeed, the patient be very old or infirm. In one case under his care, after the sloughs were separating, several attempts were made to give stimulants, but on each occasion a fresh accession of inflammation being caused, they were at length desisted from, and the man recovered very satisfactorily without. In carbuncle, as in all other diseases, the surgeon should, doubtless, pay less regard to the name and reputed pathology of the affection than to the symptoms actually present. If the system be much depressed, if the disease appear to have resulted from bad living, and, above all, if the patient's instincts demand them, stimulants should be pushed. If there be extreme restlessness, pain, exhaustion, etc., and if, after a few doses, it appears to agree with the stomach, the sustaining properties of opium may probably be very valuable. Should, however, the patient be of fair strength, the disease in its stage of advance, and attended by sharp inflammatory fever, it may be doubted whether any empirical evidence as to the usefulness of stimuli under such circumstances has yet been obtained sufficiently strong to lead the surgeon to ignore the rational principles of treatment. In such a condition, the arrest of the disease would probably be much sooner effected by alteratives. purgatives and salines, than by brandy or opium. It is even not improbable that the bleeding caused by incisions is often of great advantage.

*Local Treatment.*—From the time of Wiseman, the practice has been general of making free crucial incisions into all carbuncles. It has not, however, been absolutely universal. A few surgeons, among whom may be mentioned John Pearson in our own country, Von Walther in Germany, and Perrez in France, have been sceptical as to its efficacy. There are also in the present day some surgeons who do not recommend it. Chelius, on the contrary, who appears to have paid some attention to this question, records thus, in strong terms, his conclusion:—"That cutting into the carbuncle is generally neither useful nor necessary, is the most dangerous statement that can be put forth respecting its treatment." Respecting this point, two propositions may, we think, be maintained: 1st. Carbuncles will very frequently get well without having been incised. 2d. The practice of incision, however free, does not by any means universally arrest the carbuncular inflammation. In proof of the former, the fact, that of the cases in our list, eight were not treated by incisions, is conclusive. In these, for the most part, the patients came under care when the disease was evidently arrested, and when free openings had spontaneously formed; so that there was no object for resort to the knife. Many others besides these have been under our observation, and, as a general rule, it may be asserted that the destruction of tissues is far less extensive when the incision has been avoided, than when practiced. The consequent cicatrix is of course much less also. The assertion, that incisions, even of the freest kind, do not always arrest carbuncular inflammation, will scarcely be held to need proof by any one who has seen much of the disease. If the proof be required, however, we may refer to the fact, that in five out of the twenty-seven cases in which incisions were practiced, their repetition was subsequently necessary. Yet, granting the occasional spontaneous curability of carbuncle, and the occasional failure of treatment by incisions, the case still stands very triumphantly in favor of the latter. The only drawbacks to their use are the amount of sloughing and the large scar; but to set against these we have the complete relief of pain in almost every case, and the fact that of twenty-seven cases on our list, in no fewer than seventeen the inflammation never spread after the first incision. This latter fact is one of great importance for it cannot for a moment be held that in so large a number the cessation of the diseased action was simultaneous with the incision treatment by accident. The relation between the two must, we think, be held proven; and the fact becomes more telling when we add to it, that of the six exceptions in which the disease spread, in none did the spreading continue more than five days, and in none were more than three incisions needed. Now, it happens that a large majority of carbuncles occur in positions in which the avoidance of scar is a matter of no moment; and in those where it is important—the face, for example—the disease is so dangerous to life, that in its treatment considerations of personal beauty cannot be allowed much weight.—*London Lancet.*

